# Pregnancy outcomes among 31 patients with tetralogy of Fallot, a retrospective study

**DOI:** 10.1186/s12884-019-2630-y

**Published:** 2019-12-10

**Authors:** Kana Wang, Junguo Xin, Xiaodong Wang, Haiyan Yu, Xinghui Liu

**Affiliations:** 1 Department of Obstetrics and Gynecology, West China Second University Hospital, Sichuan University, No. 20, 3rd section, South Renmin Road, Chengdu, 610041 Sichuan China; 2 Key Laboratory of Birth Defects and Related Diseases of Women and Children, Sichuan University, Ministry of Education, No. 20, 3rd section, South Renmin Road, Chengdu, 610041 Sichuan China; 3 School of Public Heath, Chengdu Medical College, Chengdu, China

**Keywords:** Tetralogy of Fallot (TOF), Surgical repair, Uncorrected, Congenital heart disease, Pregnancy outcome

## Abstract

**Background:**

Tetralogy of Fallot (TOF) is a severe type of congenital heart disease (CHD) and it confers substantial risk to mother and fetus for pregnant women. However, the outcome of pregnancy in women with TOF has not been well studied.

**Methods:**

Women with TOF who have been seen and/or delivered at our tertiary-care hospital between April 2008 and January 2018 were retrospective reviewed.

**Results:**

A total of 31 pregnant women with TOF were identified during ten-year period. Among these patients, cardiac defects remained uncorrected in 12 women and were surgically repaired in 19 women. The frequency of miscarriages, premature birth, and the percentage of neonates of small for gestational age (SGA) were greater in the uncorrected group than the surgically repaired group (16.67% vs 0, 50% vs 5.26, 41.67% vs 10.53% respectively). The neonatal mortality and fetal mortality were not observed in the surgically repaired group, but were observed in the uncorrected group [3.23% (1/31) and 6.45(2/31) respectively]. Furthermore, the obstetric and cardiac complications in the two groups were stratified and analyzed.

**Conclusions:**

Surgical correction of TOF is associated with improved maternal and perinatal outcome. However, pregnancy in women with uncorrected TOF was still seen and it was observed at a rate of 1.4/10,000 in our medical center during ten year period. The high degree of ventricular dilatation heart, high functional classifications, serious cardiac arrhythmias and pulmonary hypertension appeared to be associated with maternal and neonatal risks.

## Background

Tetralogy of Fallot (TOF) is a severe type of congenital heart disease (CHD) with an incidence of 10% in all reported CHD, which is characterized by four components: large ventricular septal defect (VSD), overriding aorta, right ventricular hypertrophy, and right ventricular outflow tract obstruction [1]. In the past decades, the overall prognosis of CHD has been greatly improved and many patients could reach adulthood because of early diagnosis and timely therapy. However, TOF as a complex and cyanotic type of CHD, its prognosis remain poor, especially in those who did not undergo surgical repair, and it remains the leading cause of indirect maternal mortality among CHD patient with pregnancy. There are great variations in cardiac complication rates for maternal mortality among published studies for TOF patients with pregnancy, it ranged from 0 to 17.5% [2]. It has been thought that it is approximately 1/10,000 in developed countries and may be up to 25 times higher in the developing countries [3].

After surgical repair, the majority of young women with TOF could survive into their reproductive age. Without repair, TOF patients rarely reach childbearing age and get pregnant. Since pregnancy introduces extra load on the heart, and can damage cardiac functions, resulting in the increase in both maternal and perinatal morbidity [4, 5]. Previous studies have demonstrated that cardiac and obstetric complications are more likely to occur in patients without surgical repair [6–8]. The most common cardiac complications include progressive dilatation of the right ventricle and ventricular failure, thromboembolism, atrial and ventricular arrhythmias, progressive aortic root dilatation and endocarditis [1, 9]. The common obstetric complications include the increased risk in miscarriage, premature birth, and low birth weights, postpartum hemorrhage, paradoxical embolism, thromboembolism, congestive cardiac failure, infective endocarditis, and arrhythmias [6]. Among these complications, the pulmonary hemorrhage, brain abscess and thromboembolism have been thought to be the most common causes of death [1, 10].

To date, the outcomes of pregnant women with TOF have not not well characterized due to limited number of studied cases and lack of data for close follow-up. Therefore, the management of TOF pregnant women remains challenging. Here, we present 31 cases of pregnant women with or without surgical repair for TOF.

## Methods

This is a retrospective study with an approval from the Institutional Review Board of West China Second University Hospital. Between April 2008 and January 2018, a total of 85,184 pregnant women have been seen and gave birth in West China Second University Hospital. A total of 31 pregnant women with TOF were identified, and further reviewed. During analyses, we compared the overall clinical characteristics, cardiac and obstetric complications, outcome of pregnancy complications between the corrected and the uncorrected groups. In addition, we analyzed the maternal hemodynamic features and obstetric outcomes in each individual patients with uncorrected TOF.

All pregnant women with or without surgical repair were evaluated by echocardiography, electrocardiography (ECG), and routine clinical examinations such as blood pressure (BP), and heart rate (HR). Hypertension, diabetes mellitus, and other pregnancy associated diseases were taken into consideration in the clinical characterization of the patients.

### Surgical correction for TOF

Among 31 TOF pregnant patients, 12 cases underwent cardiac surgery before juvenile age (< 18 years old), and 6 cases before 25 years old. The median age for cardiac surgery was 15.52 years. The longest interval from the surgical correction to pregnancy was 29 years and the shortest was 1 year. The average interval was 11.84 years. Except one patient received two cardiac operations (cardiopulmonary correction with pulmonary artery valve replacement at 11 years before pregnancy, and atrial septal defect reparation with tricuspid valvuloplasty at 3 years before pregnancy), the remaining studied patients had one-time cardiac surgery.

### Echocardiography

During the past ten years, echocardiography for the detection of CHD and the assessment of haemodynamic status has become mature. All of our patients have been evaluated with this reliable technique. The anatomic feature of TOF including the overriding aorta, anterior deviation of the outlet septum, pulmonary stenosis and right ventricular hypertrophy have been identified and characterized by echocardiography in all studied patients [1]. The diagnosis of TOF by echocardiography has been made by the presence of a ventricular septal defect and a large overriding aorta, as well as the haemodynamic deviation for valvar functions (right ventricular pressure, ventricular dimensions, and ventricular function).

### Electrocardiogram

Arrhythmia may become manifest during pregnancy with TOF and other CHD. The 24-h ambulatory ECGs were performed in patients when they had abnormal ECG pattern or in a setting in which the patient was prone to the development of arrhythmia, such as the presence of abnormal electrolyte level.

### Pregnancy data

Data related to pregnancy in this study were mainly collected around the delivery. These data included gestational-age of newborn at birth, type of delivery, blood loss at delivery, birthweight, Apgar score and postpartum hemorrhage. Fetal and neonatal echocardiographic data have also been collected for evaluation of CHD.

### Statistics

Descriptive statistics, such as frequency, percentage, mean, standard deviation (SD), and the range were used for the presentation of variables. The normally distributed variables were presented as Means ± SD and they were compared using Student’s t-test for the differences between groups. The distribution of blood loss was presented in the format of Median ± IQR (interquartile ranges) and was compared using Mann–Whitney U test. Categorical variables, such as clinical characteristics and complications, were expressed as proportions and compared using Chi-square test or Fisher’s exact probability test. All statistical analyses were performed in SPSS (version 20.0). An alpha of 0.05 is used as the cutoff for significance.

## Results

### Patient characteristics

Thirty-one pregnant women with TOF were identified and analyzed in this study. The sociodemographic information for these patients, including age, region, education degree, gravidity and parity, were collected and summarized in the Table 1. The age of the studied patients ranged from 19 to 39 years. Specifically, the age was between 22 and 35 years (median: 28 years old) in the surgically repaired group and between 19 and 39 years (median age was 26.5 years old) in the uncorrected group. The majority of the patients who did not undergo TOF repair surgery came from remote rural areas and did not have higher education. The frequency of abortion and gestation were higher in uncorrected group than in the surgically repaired group. Of note, 94.74% (18/19) pregnant women in the corrected group were primiparity.

**Table 1:** The sociodemographic information for all studied TOF patients

Patient characteristics	Corrected (*n* = 19)	Uncorrected (*n* = 12)	*p*-Value
Age (years)	22–35	19–39	
Median age (years)	28	26.5	0.786
Region(n, %)			
city	16 (84.21)	1 (8.33)	
rural area	3 (15.79)	11 (91.67)	< 0.001
Education degree(n, %)			
with college education	12 (63.16)	0 (0)	
without college education	7 (36.84)	12 (100)	< 0.001
Gravidity(n, %)			
first	9 (47.37)	5 (41.67)	
second	3 (15.79)	3 (3.20)	
≥ three times	7 (36.84)	4 (33.33)	0.582
Parity(n, %)			
primiparity	18 (94.74)	7 (58.33)	
multiparity	1 (5.26)	5 (41.67)	0.022

The surgically procedures for TOF repair included the closure of the VSD by insertion of transannular patches, rendering the pulmonary valves incompetent, right ventricle infundibulectomy, and transannular enlargement of the right ventricular outflow tract, as detailed in the Method section. Despite of variation of the surgical procedures, the repair operations reached the goal for correction in the majority of patients, except two patients who still had residual shunt after ventricular patch.

### Cardiac and obstetric complications in two groups

Cardiac and obstetric characteristics in 31 pregnant women with TOF are summarized in Table 2 and Table 3, respectively. Obstetric and cardiac complications were observed more frequently in the uncorrected group. The rate of prematurity was significantly higher in the uncorrected group than in surgically repaired group (50% vs 5.26%, respectively, *P* = 0.007). The frequency of spontaneous abortion was also greater in the uncorrected group than in the surgically repaired group (16.67% vs none, respectively). The percentage of small-for-gestational-age newborn was 41.67% in the uncorrected group and 10.53% in the surgically repaired group (*P* = 0.078). The mean of neonatal birth weight was significantly lower in the uncorrected group than in the surgically repaired group (*P* < 0.001). However, the total days of stay in hospital and blood loss at delivery were similar in both groups (*P* = 0.866 and 0.586 respectively). The rate of postpartum hemorrhage was 10.53% in the surgically repaired group and 8.33% in the uncorrected group [due to placenta previa in both groups (2 vs 1 case, respectively), instead of abnormal blood coagulation. The CHD [Patent ductus arteriosus (PDA) and patent foramen ovale (PFO)] was observed in one baby from the surgically repaired group (Table 2).

**Table 2:** Comparisons of obstetric complications and outcome of pregnancy in TOF patients with or without surgical repair

Outcome of Pregnancies	Total *n* (%)	Corrected n (%)	Uncorrected n (%)	*p*-Value*
Total	31 (100%)	19 (61.29)	12 (38.71)	–
Miscarriages	2 (6.45)	0	2 (16.67)	0.142
Prematurity	7 (22.58)	1 (5.26)	6 (50)	0.007
SGA	7 (22.58)	2 (10.53)	5 (41.67)	0.078
LBWI	1 (3.23)	0	1 (8.33)	0.387
CHD of newborn	1 (3.23)	1 (5.26)	0	1.000
Fetal death	2 (6.45)	0	2 (16.67)	0.142
Neonatal death	1(3.23)	0	1(8.33)	0.378
Maternal death	0	0	0	–
Postpartum hemorrhage^▲^	3 (9.68)	2 (10.53)	1 (8.33)	1.000
Blood loss at delivery (ml) ^#^	31	300 (300–500)	300 (300–412)	0.674
Mean neonatal weight (kg) ^#^	28	2875 (2700–3243) (n = 19)	2060 (1202–2435) (*n* = 9)	< 0.001
HOD (day) ^#^	31	7.947 ± 3.503	7.750 ± 2.454	0.866

*P value was calculated from chi-squared test, Fisher’s exact probability, t-test or Mann whitey U; ▲Postpartum hemorrhage: blood loss > 500 ml via vaginal delivery or > 1000 ml via Caesarean section. # Data are presented as medians with interquartile ranges (IQRs). ## Data are presented as mean and range; #: data are expressed as Mean ± SD. SGA: Small for gestational age; LBWI: Low Birth Weight Infant at Term; CHD: Congenital heart defect; HOD: hospital day (total days in hospital);

**Table 3:** Comparisons of clinical characteristics, cardiac complications in TOF patients with or without surgical repair

Parameters	*n* (%)	Corrected (*n* = 19)	Uncorrected (*n* = 12)	*p*-Value*
NYHA-FC				
I-II	14 (45.16)	12 (63.16)	2 (16.67)	0.031
III–IV	17 (54.84)	7 (36.82)	10 (83.33)	
Left ventricular systolic function				
Normal	30 (96.77)	19 (100)	11 (91.67)	0.397
Dysfunction	1 (3.23)	0	1 (8.33)	
Right ventricle dilation				
Normal or mild	10 (32.26)	10 (52.63)	0	0.004
Moderate or severe	21 (67.74)	9 (47.37)	12 (100)	
Valve condition				
Pulmonary valve stenosis	16 (51.61)	4 (21.05)	12 (100)	< 0.001
Pulmonic regurgitation	6 (19.35)	6 (31.58)	0	0.059
Tricuspid regurgitation	13 (41.94)	10 (52.63)	3 (25)	0.158
Mitral regurgitation	1 (3.23)	1 (5.26)	0	1.000
Arrhythmias				
normal	5 (16.13)	5 (26.32)	0	0.128
Right ventricular hypertrophy	17 (54.84)	5 (26.32)	12 (100)	< 0.001
CRBB	9 (29.03)	9 (47.37)	0	0.005
IRBBB	6 (19.35)	3 (15.79)	3 (25.00)	0.653
Sinus tachycardia	3 (9.68)	2 (10.53)	1 (8.33)	1.000
I°AVB	2 (6.45)	1 (5.26)	1 (8.33)	1.000
APB	1 (3.23)	1 (5.26)	0	1.000
VPB	2 (6.45)	2 (10.53)	0	0.510
Pulmonary hypertension	1 (3.23)	0	1 (8.33)	0.387
Aortopulmonary collateral vessels	3 (9.68)	0	3 (25.00)	0.049
Pericardial effusion	2 (6.45)	0	2 (16.67)	0.142

*P value was calculated from chi-squared test, Fisher’s exact probability, t-test or Mann whitey U; NYHA-FC: cardiac function grading (New York Heart Association); CRBB: complete right bundle branch block; IRBBB: incomplete right bundle branch block; AVB: atrioventricular block; VPB: ventricular premature beat; APB: atrial premature beat

Most of patients in the surgically repaired group had good cardiac functional status with NYHA class of I-II, which was significantly better than uncorrected group (63.16% vs 16.67%, respectively, *P* = 0.031). Ten (83.33%) cases had cardiac function NYHA class III or higher in the uncorrected group. Cardiac diameter and ventricular systolic function were assessed in all patients. The moderate or severe right ventricle dilation was found in all uncorrected patients (12 cases, 100%), but only in 9 cases (47.37%) in the surgically repaired group. The right ventricular dilation was absent or in a very mild degree in the remaining 10 corrected cases (52.63%) (*P* = 0.004). Of note, one uncorrected patient showed a limited systolic function (EF = 31%, FS = 16%) and pulmonary arterial hypertension (PAH). In the current study, the most common complications attributable to TOF were outflow tract and valve condition, ventricular shunting with ventricular septal defect and arrhythmias. Pulmonary valve stenosis was seen in all uncorrected patients (100%) and in 4 cases (21.05%) of corrected TOF (*P* < 0.001). Pulmonic regurgitation was found in 6 (31.58%) in the surgically repaired patients but none in uncorrected patients (*P* = 0.059). According to the ECGs, ventricular hypertrophy was present in all uncorrected patients (100%) but only 5 cases in surgically repaired groups (26.32%, *P* < 0.001). Following surgical repair, ECG became normal in 5 patients (26.32%), but complete right bundle branch block (CRBB) have been still detected in 9 (47.37%) corrected patients. No other abnormal ECG was found between two groups (Table 3).

### Maternal and perinatal outcomes in the women with uncorrected TOF

The detailed clinical information concerning maternal and perinatal outcomes in the women with uncorrected TOF is summarized in Table 4. Among them, five women (41.67%) were primigravidae (G1P0), the remaining 7 women had a history of abortion or childbirth. Except that one patient with intrauterine fetal death who underwent transvaginal complete curettage of uterine cavity, the mode of delivery in remaining patients was cesarean section (C-section). During the operation, 6 patients were under general anesthesia, 4 patients under epidural anesthesia, and 1 patient under combined spinal-epidural anesthesia. Regarding the obstetric complications related to the prematurity including miscarriages, premature labor, small for gestational age (SGA) and low birth weight infant at term (LBWI), half of women (50%) were due to premature rupture of fetal membranes (PROM) and/or prenatal hemorrhage. The rate of SGA was 41.67% (*n* = 5). One case with NYHA class IV complicated with severe pulmonary hypertension and limited systolic function had to terminate pregnancy at 27 + 6 weeks. That patient was transferred to the Department of Cardiology for further treatments and survived. Unfortunately, her baby died at the 7th day after birth due to neonatal pneumonia, septicemia and intracranial hemorrhage. The maternal mortality in this study was none. Neonatal mortality and fetal mortality were observed only in the uncorrected group with a rate of 3.23% (1/31) and 6.45% (2/31), respectively (Table 4).

**Table 4:** Maternal Hemodynamic Features and Obstetric Outcomes in Pregnant Woman with Uncorrected Tetralogy of Fallot

#	G/P	NYHA-FC	RVdiameter (mm)	RVAW (mm)	RV outflow tract	PA (mm)	VSD (mm)	Other	LVSD (%)	Arrhythmia^▲^	Obstetric complication	Mode of delivery	Anaesthesia	Outcome
1	G5P1	III	30	5	4	12	20	^a^Collat. circ.	EF = 72 FS = 40	–	SGA	Caesarean section: 38 + 3 weeks	General anaesthesia	M – good N – live, 2500 g
2	G1P0	II	31	9	17	15	16	ASD TR PDA	EF = 80 FS = 36	–	Prematurity	Caesarean section: 35 + 6 weeks	General anaesthesia	M – good N – live, 1935 g
3	G5P2	III	35	10	11	14	–	–	EF = 50 FS = 28	I°AVB	SGA,Prematurity	Caesarean section: 35 weeks	Epidural analgesia	M – good N – live, 1160 g
4	G1P0	III	33	10	9	17	20	–	EF = 60 FS = 30	–	Prematurity	Caesarean section:33 weeks	General anaesthesia	M – good N – live, unknown
5	G8P1	III	30	13	15	24	20	–	EF = 58 FS = 31		Prematurity	Caesarean section:36 + 1 weeks	General anaesthesia	M – good N – live, 2950 g
6	G2P1	II	28	10	11	22	22	–	EF = 56 FS = 29		SGA, LBWI	Caesarean section:38 weeks	Epiduralanaesthesia	M – good N –live,2070 g
7	G3P2	IV	30	14	13	17	17	PH ^a^Collat. circ.	EF = 31 FS = 16	–	Prematurity	Caesarean section:27 + 6 weeks	General anaesthesia	M – good N –1245 g, died at post-partum
8	G2P0	III	36	12	8	11	15	Right aortic arch	EF = 58 FS = 29	IRBBB	Prematurity	Caesarean section: 34 + 2 weeks	Epiduralanaesthesia	M – good N – live,unknown
9	G1P0	III	38	11	9	15	17	^a^Collat. circ.	EF = 58 FS = 30	Sinus tachycardia	SGA	Caesarean section:36 + 6 weeks	Combined spinal epidural	M – good N – live,2200 g
10	G1P0	III	37	9	10	18	22	TR	EF = 70 FS = 38	IRBBB	Miscarriages	Vaginal delivery: 9 weeks	unknown	M – good N –Stillbirth
11	G2P0	III	24	10	8	8	22	ASD	EF = 67 FS = 36	–	Miscarriages	Caesarean section:16 + 6 weeks	Epiduralanaesthesia	M – good N –Stillbirth
12	G1P0	III	27	10	8	13	14	Right aortic arch	EF = 67 FS = 36	IRBBB	SGA	Caesarean section:37 weeks	General anaesthesia	M – good N – live, 2060 g

*G/P* Gravidity and parity history, *NYHA-FC* cardiac function grading (New York Heart Association), *RV* Rightventricular, *PA* pulmonary artery, *RVAW* right ventricular anterior wall, *VSD* ventricular septal defect, *ASD* atrial septal defect, *PDA* Patent ductus arteriosus, *TR* tricuspid regurgitation, *PH* Pulmonary hypertension, *LVSD* left ventricular systolic function, *AVB* atrioventricular block, *IRBBB* incomplete right bundle branch block, *SGA* Small for gestational age, *LBWI* Low Birth Weight Infant at Term

^a^Collat.circ.: Aortopulmonary collateral vessels; Arrhythmia^▲^:All patients showed right ventricular hypertrophy

## Discussion

Tetralogy of Fallot is a severe type of CHD and it is characterized by the hemodynamic alterations due to anatomic abnormality, with varied degree of comprised cardiac functions, including ventricular dysfunction, right ventricular systolic dysfunction, right ventricular dilation, outflow tract obstruction, and pulmonary hypertension [11]. After surgical repair of TOF, the majority of young women could survive into their reproductive age. But it is generally known that heart disease constitutes a leading nonobstetric cause of maternal mortality, especially in patients with the cyanotic and complex shunt lesions [6]. Previous studies for pregnant women with TOF showed that pregnancy confers a considerable risk to these patients. The adverse cardiovascular events may be associated with right ventricular dysfunction, severe pulmonary hypertension, and severe pulmonic regurgitation with RV dysfunction [3, 11]. It has been demonstrated that abnormal uteroplacental Doppler flow (UDF) was associated with right ventricular function parameters, suggesting that maternal cardiac dysfunction contributes to defective placentation and/or placental perfusion, which subsequently increases the incidence of obstetric and neonatal complications [12].

With emerging more advanced diagnostic and therapeutic approaches, the overall prognosis of TOF patients has been remarkably improved in the past decades. However, we occasionally encountered that women with uncorrected TOF got pregnant. Due to the extra cardiac load during pregnancy, women with uncorrected TOF had much higher maternal and perinatal morbidity compared to general population [1–5, 9]. These cases are relatively rare and almost all of published studies for these patients in the literature were case reports. Thus far, no standard regimen has been established to manage these patients. Accordingly, it will be extremely beneficial to study these cases in a comprehensive manner. This study was for the first time to collect over 30 cases of TOF patients from a single center and perform comparative analysis between uncorrected cases and surgically repaired cases.

It is worthwhile to stress that right ventricular dilation were found in all uncorrected patients but in less than half patients in the surgically repaired group. In addition, no incidence of thromboembolism and endocarditis were found in studied subjects, which may explain the overall good clinical outcomes in spite of different degrees of arrhythmias. Women with NYHA class > III have a relatively poor prognosis during pregnancy [13]. Of note, one uncorrected patient with NYHA class IV was the only patient with limited left ventricular systolic function and pulmonary arterial hypertension. The pregnancy was terminated in that patient at 27^+ 6^ weeks due to severe ventricular failure. The data obtained from the current study collectively implied that the high degree of right ventricular dilation and high NYHA classification are the most relevant factors for negative outcomes.

It has been demonstrated that women at childbearing age with surgical repair for TOF have lower pregnancy-related risks, and the changes in ventricular dimensions and NYHA class are consistent with normal pregnancy adaptation [14]. In the literature, it has been demonstrated that pregnant patients even with corrected TOF still have a higher risk and poorer outcome than otherwise healthy women. The increased risk may be attributed to the extra haemodynamic burden and exacerbation of residual cardiovascular lesions, or recurrence of right ventricular outflow obstruction, right ventricular dilatation, pulmonary regurgitation, the right ventricular dilatation and failure, as well as atrial and ventricular arrhythmia [15–17]. In the current study, the residual shunt at VSD patches were present in two patients following operations and their NYHA class were grade III. In addition, 6 patients (31.58%) had pulmonary regurgitation and about two-thirds of patients had arrhythmia at different degrees. Our data support the notion that pregnant women with the surgically repaired TOF are still in high risk, depending on the degree of the cardiac functional adaption during pregnancy.

Regarding the functional adaption of pregnant women with corrected TOF, Egidy et al monitored the quantitative volumetric changes, and concluded that that those women with successful pregnancy appeared to experience an accelerated rate of right ventricular remodeling (an increase in end-diastolic volume) [14]. In our study, the moderate or severe right ventricular dilation was found in all of uncorrected patients (100%), but in less than half of the patients in surgically repaired group (47.37%). In addition, the right ventricular hypertrophy and pulmonary valve stenosis were also been found in all of uncorrected patients (100%), but much less in the surgically repaired patients. The comparisons between the corrected and uncorrected patients revealed that the risk of the women with corrected TOF might be divided to two groups, high-risk and low-risk patient groups according to the degree of right ventricular dilation/hypertrophy and the pulmonary valve stenosis. Further study with a large number of cases that are sufficient to stratify data for high-risk and low-risk patients is necessary to define their actual prognostic value.

Maternal condition and mortality has significant effects on fetal outcome in CHD patients [18]. Recently, Ramage et al. reported 2114 births to women with adult congenital heart disease (ACHD) and suggested an association between several adverse neonatal/maternal outcomes and ACHD [19]. Their results showed that preterm births (< 37 weeks gestation) were 1.4 times higher in women with ACHD than those without ACHD. Women with ACHD also had a higher odd of having a preterm birth with a gestation less than 32 weeks. In addition, 12.8% women with ACHD and 8.7% of women without ACHD delivered an SGA infant. Consistent with the study conducted by Ramage, the complications were more frequently present in the uncorrected group in our study. Although all of the patients have survived, most all of them suffered cardiac and obstetric complications at varying degrees. The low maternal cardiac output, intrauterine growth restriction and the SGA may occur even after the surgical repair of TOF [20].

In consideration of high complication risks, woman with cardiac disease may be safer with a C-section delivery to avoid prolonged laboring time [21]. In this study, one patient with intrauterine fetal death undergone transvaginal complete curettage of uterine cavity, other uncorrected patients delivered by C-section successfully. Women at highest risk may benefit from preconception counseling and close clinical monitoring during pregnancy [19]. Careful interdisciplinary management among the cardiologist, obstetrician, anesthetist, and neonatologist, and detailed plans for delivery may improve the prognosis [22, 23]. Although the differences of outcomes between 19 surgically repaired and 12 uncorrected cases were significant, the impact of other clinical parameters was not achieved in the current study due to the small number of cases in the subgroups. Further research with a large number of cases to determine the relationship between the outcomes and other clinical parameters may be helpful to establish more beneficial management regimen for TOF patients, especially for those without surgical repair. In addition, it will be beneficial to study the each pregnancy outcome of these TOF patients (with or without repair) in instead of per woman. However, it has not been performed in the current study due to the limitation to retrieve complete medical record for previous pregnancies and no record for new pregnancies in these patients.

## Conclusions

In summary, TOF confers a high-risk of cardiac and obstetric complications for pregnant women, especially in those who did not received repair surgery. However, due to economic and regional constraints, patients who did not have a chance to receive timely surgical repair and got pregnancy were occasionally seen in our practice. In the past ten years, 12 such cases among 85,184 pregnancies (1.4 out of 10,000) was seen in our medical center. From our experience, these patients may still be able to have a successful pregnancy, but exclusively depending on professional care from multidisciplinary teams. In addition, while the exact implications of clinical parameters remain to be defined with a larger number of cases, the high degree of ventricular dilatation heart, high functional classifications, serious cardiac arrhythmias and pulmonary hypertension appeared to be associated with maternal and neonatal risks in the patients studied in the current investigation. Routine cardiac examination should be performed before pregnancy to exclude possible cardiac diseases and the cardiac surgery should be performed early. For pregnant women with TOF, close monitoring should be strengthened no matter whether they have received surgical repair or not. Decision for the mode of delivery should be individualized by weighing the risks and benefits in a given clinical situation [2, 23].

## Data Availability

The raw data supporting this study can be requested via the corresponding author.

## Abbreviations

ACHD: Adult congenital heart disease
APB: Atrial premature beat
AVB: Atrioventricular block
BP: Blood pressure
CHD: Congenital heart disease
CRBB: Complete right bundle branch block
CS: Caesarean section
ECG: Electrocardiography
HOD: Hospital day (total days in hospital)
HR: Heart rate
IRBBB: Incomplete right bundle branch block
LBWI: Low birth weight infant at term
NYHA-FC: Cardiac function grading (New York Heart Association)
PAH: Pulmonary arterial hypertension
PDA: Patent ductus arteriosus
PFO: Patent foramen ovale
PROM: Premature rupture of fetal membranes
SGA: Small for gestational age
TOF: Tetralogy of fallot
UDF: Uteroplacental doppler flow
VPB: Ventricular premature beat
VSD: Ventricular septal defect
